# 

**Published:** 1888-07-21

**Authors:** 


The electric light very much resembles sunlight; it is rich in the violet or chemical rays, and numerous experiments have proved that plants grow and fruits ripen under its influence. The fear has been expressed that if this means of illumination were to become general we might wear out too soon. This only time can show. It has not yet been proved, however, that we run down more quickly in the summer than in the winter, nor yet that, in addition to our ordinary slumber, a period of hibernation is necessary to the well-being of those who dwell in the more northern climes.

Theory versus Practice.—Waiter in American hotel: "You are late for lunch, sir." Eminent Physician: "Yes, I had to finish my magazine article on "The Laws of Health," so as to catch the post. What have you to-day?" Waiter: "Hot rolls, clams, plum pudding, apple-dumplings, mince pie, and fruit cake." Physician: "Bring them all."

**Special Notice to Newsagents, Advertisers, and the Trade.**

**CHANGE OF OFFICE AND PUBLISHER.**

All business communications should be addressed to the Manager, Office of The Hospital, 38, Old Jewry (Cheapside end). All Cheques and Post Office Orders should be made payable to J. H. S. Hanning, Secretary, 38, Old Jewry, E.C., crossed Lloyds, Barnetts, and Bosanquet's Bank (Limited).

**To Secretaries and Matrons.**

The Editor will be glad to have a copy of the Regulations, and also of every Report issued from the Press of Asylums, Hospitals, Convalescent and Nursing Institutions, as well as those of other Charities, so soon as they are published, and an early copy in duplicate of all appeals for funds. Notices of Annual and other Meetings, Festival Dinners, etc., will be welcomed. All such communications should be addressed to *The Editor, The Lodge, Porchester Square, London, W.* Any superintendent, secretary, matron, or other chief official who will regularly send such documents and information will be registered to receive free of cost a cover for binding The Hospital in half-yearly volumes on sending name and address to the publisher.

"THREE scruples make a dram," is taught
By teachers to their pupils;
Yet drams if they're too often sought
Won't add unto your scruples.—*Judge.*

HAD a choice of the manner of his death been offered to Mr. Matthew Arnold, it is probable that he would have chosen no other than the swift fashion in which he passed from among men: at least so we may surmise from his poem called "The Wish," in which he indicates the sort of death-bed he desires. One stanza of it is not flattering to the medical profession:

"Nor bring to see me cease to live
Some doctor full of phrase and fame,
To shake his sapient head, and give
The ill he cannot cure a name."

### AMUSEMENTS WITH PROFIT FOR ALL AGES.

**Rules.**—All answers must be addressed to the PRIZE EDITOR, THE HOSPITAL, 38, Old Jewry, Cheapside, London, E.C., not later than the *first* post on Thursday next. The full name and address must in all cases be given, but a *nom de plume* may be added for publication. Only one side of the paper must be written on.

### FOR MEN AND WOMEN.

### First Quarterly Poetical Competition.

Commenced May 26th, 1888; ends August 25th, 1888.

This quarter a PRIZE of ONE GUINEA will be given to the competitor who gains the highest number of marks for original poems, on any subjects suitable for insertion in this paper. All contributions sent in will be credited according to merit, eighteen marks being the highest score for any one contribution. This competition to alternate weekly with the set acrostic competition.

### Acrostic Competition.

This quarter the original acrostic competition will be discontinued, and A PRIZE of HALF-A-GUINEA will be given to the competitor who gains the highest score for solution of acrostics set. *One mark* only will be given to the competitors who solve all the lights of each acrostic.

Exception will be made in the case of *quotation acrostics*, when *two marks* will be given, *one* for the correct solution of all *lights*, and *one* mark for the *source* of the quotations.

This week credit will be given for solution of

### No. 4.—Double Acrostic.

" In happy hour hath he received
The laurel, meed of famous bards of yore,
Which Dryden and diviner Spenser wore."

1. " His honour rooted in dishonour stood,
And faith unfaithful kept him falsely true."
2. " The minstrel of that ancient name—
Was none who struck the harp so well
Within the Land Debateable."
3. " He sleeps upon his craggy bed."
4. " How sharper than a serpent's tooth it is
To have a thankless child!"
5. " The mighty sorrow hath been borne,
And she is thoroughly forlorn;
Her soul doth in itself stand fast,
Sustained by memory of the past."
6. " Peace, peace! He is not dead—he doth not sleep—
He hath awakened from the dream of life."
7. " 'Twas his
In life and death to be the mark, where wrong
Aimed with her poisoned arrows—but to miss,
O Victor unsurpassed in modern song!"
8. " The mantle's fold
In many an artful plait she tied,
To show the form it seemed to hide."

### Answers.

### No. 3.—Double Acrostic.

S pellbaun D
L ilywhit E
U nbelie F
M elinit E
B rito N
E mpiri C
R espit E

Correct answers have been received from Condorcet.

### THE WORD COMPETITION.

Third Quarterly Competition commenced

May 5th, 1888; ends August 4th, 1888.

Three prizes of one guinea, 15*s.* and 10*s.* 6*d.* will be given each quarter to the three competitors who obtain the highest number of marks by making the largest number of words derived from the word or phrase set for anatomical dissection; that for this, the *eleventh* week of the quarter, being

" ITALIAN."

OBSERVE.—Proper names, abbreviations, foreign words, words of less than four letters, and repetitions are barred; plurals, and past and present participles of verbs are allowed. Nuttall's *Standard* dictionary only to be used.

N.B.—All words sent in must be arranged *alphabetically*, with correct total affixed.

### Results of Ninth Week.

*Universal*—Time (244), Patience (241), Lightowlers (231), Dodo (230), Lauriston (229), Sym (213), Alicia (208), Reldas (201), Puff 175).

### Notices to Correspondents.

*A. M. E.*—Yes. *O. P.*—Twelve marks.

### THE CHILDREN'S CORNER.

PRIZES.

FOR MOST CORRECT ANSWERS TO PUZZLES.

This Commenced October 1st, 1887.

One Pound a Month.

A PRIZE OF THREE GUINEAS

to the Competitor who shall have sent in the largest number of correct or best answers during the twelve months.

### Puzzles.—Third Week.

No. 9. HISTORICAL CHARADE.—My first and second give the name of oaten cake, much eaten in Scotland; my third is what my former should not do in baking; my whole gives the name of a battle gained by the Scots against the English.

No. 10. DOUBLE ACROSTIC.

One crossed the desert of the rolling western wave,
One pierced to winter's inmost hold, to fill an unknown grave.

1. I dry the small boy's tears, I make them flow.
2. Pheasants and filberts all aglow.
3. Lo! where in broiling stream it rushes past.
4. I once held ashes, now hot water fast.
5. This all who value peace should be when angry passions roar.
6. A little piece of paper or a weapon used in war.
7. Region of lakes and towering summits white.
8. A mighty king in whom four crowns unite.

No. 11. DECAPITATION.—I am a monster of the deep; behead me, I am an exclamation; behead me again and I am a chest.

No. 12. CHARADE.

My *first* is a bird that early awakes,
My *second* is found in rivers and lakes;
My *whole* is an insect as black as a rook,
Which is often a pest to the baker and cook.

### Results of First Week's Puzzles.

No. 1. DOUBLE ACROSTIC.

H andica P
O dont O
O vertur E
D eligh T

No. 2. RIDDLE-ME-REE.—Horse.

No. 3. GEOGRAPHICAL CHARADE.—West-more-land.

No. 4 ENIGMA.—A drum, and the drum of the ear.

*Four marks each.*—Gem. Scrooge. *Three marks each.*—Thanet, Poodle, Boadicea, A.C.K., Jack, Curly. *Two marks each.*—Alsie, Young Surgeon, Hercules, Geranium.

### Conditions.

I. Every paper sent in must be clearly written, and one side only must be used. All competitors must mark at the head of their papers the number of their competition.

II. Each paper must be signed by the author with his or her real name and address. A *nom de plume* may be added, if the writer does not desire to be referred to by us by his real name. In the case of all prizewinners, however, the real name and address will be published.

III. All communications relating to prize competitions should be addressed to "The Prize Editor, THE HOSPITAL, 38, Old Jewry, London, E.C." Competitors are requested not to include in letters, etc., to the Prize Editor communications on matters of other business. All letters must be fully prepaid.

IV. The Prize Editor of THE HOSPITAL is the sole judge of the competitions, and his decision is in all cases final and without appeal.

V. The Prize Editor reserves the right to publish any paper sent in, whether it receives a prize or not. He does not hold himself responsible for any MSS. sent, nor can he undertake to return rejected competitions.

VI.—All children resident at school are requested to forward their *home* address in addition to the school one, when entering for the "Children's Corner" Competitions.

**Notice to all Correspondents.**—N.B.—All letters referring to this page, which do not arrive at 38, Old Jewry, Cheapside, London, E.C., by the *first post on Thursdays*, and are not addressed PRIZE EDITOR, will in future be disqualified and disregarded.

No one will be permitted to win the Monthly Prizes twice in any one year, the Annual Prize being offered especially to prevent this.

